# Diethylstilbestrol 1 mg in the Treatment of Acute Urinary Retention due to Prostatic Obstruction in the Elderly: A Preliminary Study

**DOI:** 10.1155/2014/984382

**Published:** 2014-01-19

**Authors:** Leonardo Oliveira Reis, Gustavo Borges De Mendonça, Bruno D. Carneiro, Edson Schneider, Eduardo Varella Gewehr, André Meirelles, Fernandes Denardi, Antonio Gugliotta

**Affiliations:** ^1^Faculty of Medicine, Pontifical Catholic University of Campinas (PUC-Campinas), 13060-904 Campinas, SP, Brazil; ^2^Urology Division, Pontifical Catholic University of Campinas (PUC-Campinas), 13060-904 Campinas, SP, Brazil; ^3^Faculty of Medicine and Urology Division, University of Campinas (Unicamp), 13083-887 Campinas, SP, Brazil

## Abstract

Patients who failed a catheter-free trial after acute urinary retention and one week of full dose alpha-blocker and 5-alpha-reductase inhibitor were offered Diethylstilbestrol 1 mg plus Aspirin 100 mg over 4 weeks. Prostate volume, age, serum creatinine, and initial retention drained urine volume were recorded. After excluding cardiovascular morbidity (*n* = 7), upper urinary tract dilation (*n* = 3), compromised renal function (*n* = 2), urinary tract infection (*n* = 2), neurological diagnosis (*n* = 2), or preferred immediate channel transurethral resection of prostate (*n* = 5), 48 of 69 consecutive patients ≥70 years were included. Mean age was 76.6 years (70–84), mean prostate volume 90 cm^3^ (42–128), and mean follow-up 204 days; 58% (28/48) were passing urine and 42% (20/48) were catheter dependent after 4 weeks Diethylstilbestrol trial. Mean age and drained urine volume of catheter dependent patients were 82.4 years and 850 mL compared with 74.6 years and 530 mL in catheter-free men, respectively. Age and drained urine volume were independent predictors of catheter-free trial (both *P* < 0.01). Seventy-five percent (6/8) of patients 80 years and older were catheter dependent. Transient nipple/breast tenderness and gynecomastia were the only adverse effects reported by 21% (10/48) and 4% (2/48), respectively. No patient presented severe complications.

## 1. Introduction

Acute urinary retention (AUR) is an important urological emergency in men [1–3]. It is characterized by a sudden and painful inability to pass urine voluntarily [1, 4]. Approximately 10% of men aged 70 and a third of men aged 80 will have an AUR in the next 5 years [5].

Management of AUR consists of immediate bladder decompression by catheterization. The method and duration of catheterization as an initial treatment are primarily dependent within and among the countries, as is the decision to admit or send home after catheterization [1, 6, 7]. Usually, standard management of nonhigh pressure acute retention is alpha-blocker for at least 48 hours followed by trial without catheter (TWOC) [6, 8, 9].

Subsequent handling is not well filed due to lack of guidelines, mainly in those who fail TWOC [1]. This study aims to evaluate efficacy, safety, and adverse effects of DES 1 mg plus aspirin 100 mg over 4 weeks as a minimally invasive alternative in an elderly population presenting with AUR secondary to prostatic obstruction who failed a catheter-free trial.

## 2. Methods 

After local ethics committee approval and patient consent, men over 70 years, age in an outpatient clinical setting presenting prostatic obstruction with AUR and who after one week of full dose alpha-blocker and 5-alpha-reductase inhibitor have failed a catheter-free trial were invited to be enrolled in a prospective, uncontrolled study to establish the rate of catheter dependence and its predictors in elderly men after diethylstilbestrol (DES) 1 mg plus aspirin 100 mg over 4 weeks. Catheter-free trial was defined as resolution of AUR after bladder decompression by catheterization.

All patients underwent urinalysis, ultrasonography accessing prostate volume, bladder and upper urinary tract and laboratorial work up including serum creatinine. Drained urine volumes were recorded at the first catheterization. The primary end point was the number of catheter-free versus catheter dependent patients.

Exclusion criteria were cardiovascular morbidity, compromised renal function measured by abnormal creatinine levels, hematuria, bladder stone, upper urinary tract dilation, urinary tract infection, neurological diagnosis, DES or aspirin intolerance/contraindication, and prostate cancer diagnosis or suspicion.

Multivariate analysis was utilized to identify independent predictors of TWOC and the level of significance was *P* < 0.05.

## 3. Results

Among 69 patients screened, 16 were excluded due to cardiovascular morbidity (*n* = 7), upper urinary tract dilation (*n* = 3), compromised renal function (*n* = 2), urinary tract infection (*n* = 2) and neurological diagnosis (*n* = 2).

The proposed protocol was offered to 53 consecutive patients, 5 preferred immediate channel transurethral resection of prostate (TURP) and 48 concluded the trial. Two patients underwent prostate biopsy to rule out prostate cancer before trial enrollment.

Among the analyzed patients, mean age was 76.6 years (70–84), mean prostate volume 90 cm^3^ (42–128), and mean follow-up 204 days; 58% (28/48) were passing urine and 42% (20/48) were catheter dependent after 4 weeks DES trial.

Mean age and initial retention drained urine volume of catheter dependent patients were 82.4 years and 850 mL compared with 74.6 years and 530 mL in catheter-free men, respectively. Age and drained urine volume were independent predictors of TWOC (both *P* < 0.01). Seventy-five percent (6/8) of patients 80 years and older were catheter dependent.

Transient nipple/breast tenderness and gynecomastia were the only adverse effects reported by 21% (10/48) and 4% (2/48), respectively. No patient presented severe complications.

## 4. Discussion

Considering the morbidity of long-term catheter and the fact that some patients refuse surgical treatment, advancement of prostatic obstruction medical treatment is merited.

Offering TURP to a patient must be placed in the context of their anesthetic risk, wishes, and alternative strategies. At the same time, prolonged use of indwelling catheter (IDC) is accompanied by several side effects and complications [10]. In addition, the cost implication of having an IDC is considerable [11].

In this context, clean intermittent self-catheterization (CISC) adds the convenience of not having an external device and the maintenance of sexual activity. About half of the patients using CISC will eventually void spontaneously and the incidence of urinary tract infection is half of that with IDC even after TURP. However, CISC requires a dedicated staff to efficiently teach the method, and patients able to manage CISC tend to be younger and have smaller prostates [12].

Previous proof of concept and safety of hormonal treatment for prostatic obstruction was presented for patients with prostate cancer and AUR [13, 14] and also short-term hormonal treatment for prostatic obstruction in a noncancer scenario [15, 16].

Considering the increasingly aging population and that less surgical morbidity, longer life expectancy, and higher impact of hormonal treatment in terms of quality of life and collateral effects are expected among youngers, the elderly were selected to this preliminary protocol.

Aiming acceptable adverse effects and risk-benefit balance, hormones were discontinued after 4 weeks and also antiplatelet was added to treatment. Besides, the local ethics committee encouraged this preliminary trial before advancing. Additionally, 5 alpha reductase inhibitor plus alpha blocker were continued indefinitely supposedly to limit the likely decrease in the initial success rate with time.

Based on the safety and relatively low morbidity of the current study, longer trials of hormones are warranted and would add to this modest 58% medium term success rate. On the other hand, if confirmed in larger trials with longer follow up, it is promising the fact that more than half the patients were initially rescued after having failed a TWOC.

Moreover, considering surgical hemorrhagic complications, medical hormonal treatment may be a potential (definitive or palliative) alternative to patients under oral anticoagulation once it precludes cardiovascular morbidity and at the same time increases surgical morbidity.

In case of incomplete response, the proposed alternative not only refers to how to avoid any surgical treatment, but also allows how to achieve a better condition for a future surgical procedure in a catheter-free patient, avoiding bladder bacterial colonization and its potential consequences once surgery morbidity for catheterized patients is potentially risky compared to those catheter free when treated [10].

Varenhorst and Alund have described 65.6% (80/122) catheter-free rate six months after starting endocrine therapy in prostate cancer patients under AUR. The mean period of catheterization for the 80 patients who responded was 2.7 months after orchiectomy and 3.4 months during estrogen treatment [13].

Also, in prostate cancer patients not candidates for curative treatment, androgen deprivation therapy improved lower urinary tract symptoms, objective voiding parameters, and prostate volume in a clinically relevant manner within the first month [14], supporting the effect of short-term treatment observed in the present study.

Even examining patients without cancer diagnosis, Narayan et al. described that flutamide reduced the prostate volume in a dose-related fashion and resulted in an increase in peak flow rate at 4 weeks (250 mg three times daily, *P* value < 0.05) [15] and Onu showed the effects of depot medroxyprogesterone 150 mg single-dose intramuscular injection in a similar fashion, as a safe and effective treatment for prostatic obstruction where potency is a secondary consideration [16].

Advanced age and higher drained urine volume as predictors of treatment failure in the present study denote chronic retention with poor detrusor function (elderly bladder) that represents risk factors of both surgical and conservative therapy failure [17, 18] and that should be confirmed in future studies. However, although the present study failed to include bladder functional evaluation, it would add important morbidity given the substantial invasiveness of urodynamic study, mainly in the elderly.

Furthermore, patients reported good tolerance, which could be related with elderly “natural” hormonal decline, where sexual function is a secondary consideration. Future studies should consider erectile function and testosterone level dosages before and after treatment.

Although to the best of our knowledge, this is the first report of DES to rescue a selected elderly population presenting with AUR who failed a catheter-free trial, it is important to highlight that this is a preliminary study with limitations such as no data regarding detrusor function (urodynamic study) or contributors to impaired detrusor function (fecal impaction and medications), relatively small number of patients included, no distinction between spontaneous and precipitated AUR, relatively short follow-up, the lack of control arm (i.e., a second trial without catheter after continuous full dose alpha-blocker and 5-alpha-reductase inhibitor over 4 weeks), no data regarding PSA, and testosterone and prostate volume variations during the study.

Finally, although 32% of the patients with successful TWOC will require surgery within 8 to 24 months of follow-up [19], the good results reported might be related to the exclusion criteria that may input selection bias for those with better bladder function, reducing the generalizability of the data.

## 5. Conclusion

Low dose diethylstilbestrol associated with aspirin seems to be a safe and effective minimally invasive alternative to rescue a significant portion of a selected elderly population presenting with AUR secondary to prostatic obstruction who failed a catheter-free trial after one week of full dose alpha-blocker and 5-alpha-reductase inhibitor and refuses immediate channel TURP.

Higher drained urine volume and advanced age were associated with higher failure. Overall, patients reported good tolerance. Larger trials with longer follow-up are warranted before systematic utilization of hormonal therapy is anticipated in this scenario.
